# Sexual Dimorphism in Renal Progenitors: Do Immunosuppressants Erase the Female Advantage?

**DOI:** 10.3389/ti.2026.16092

**Published:** 2026-03-10

**Authors:** Zeynep Ural

**Affiliations:** Department of Nephrology, Lösante Hospital, Ankara, Türkiye

**Keywords:** estrogen, immunosuppression, kidney transplantation, renal progenitors, sex differences

Dear Editors,

Sex-based differences in chronic kidney disease (CKD) progression are well recognized: women generally experience slower renal function decline and greater resistance to podocyte injury than men. Paradoxically, this biological advantage largely disappears after kidney transplantation, where graft outcomes become broadly comparable between sexes [1]. The mechanisms underlying this loss of sexual dimorphism remain poorly understood.

Recent mechanistic studies on estrogen-regulated renal progenitors offer a unifying explanation [2]. Estrogen exerts renal protection through two complementary molecular axes. First, estrogen activates the ERα–PI3K/AKT–mTOR pathway, promoting renal progenitor proliferation and podocyte renewal, counterbalancing podocyte loss and preserving glomerular integrity. Elegant lineage-tracing and functional studies demonstrate that estrogen-dependent progenitor activation contributes directly to female renal resilience and adaptive capacity [3–6]. Second, estrogen physiologically suppresses calcineurin–NFAT signaling, limiting pro-inflammatory transcription, cytoskeletal destabilization, and maladaptive hypertrophy in podocytes [7, 8]. Together, these pathways establish a coordinated balance between regeneration and immune restraint, forming the biological basis of female renal advantage (Figure 1).

**FIGURE 1:**
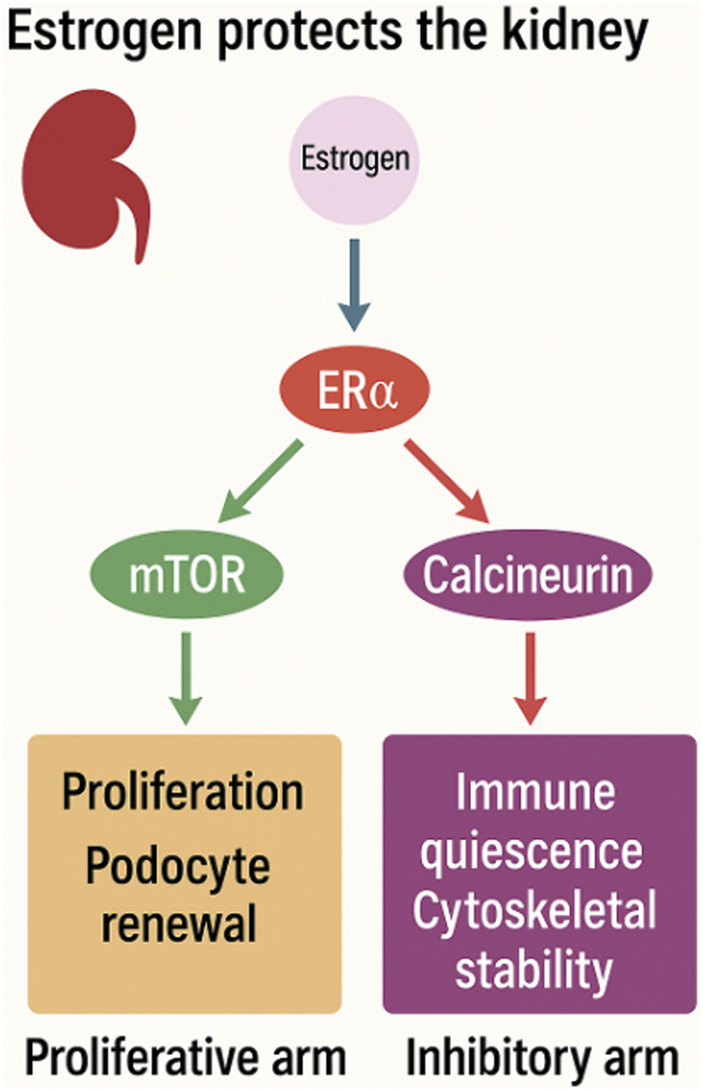
Estrogen protects renal integrity by coordinating dual molecular pathways.

Testosterone, by contrast, drives these same pathways toward maladaptive outcomes. It stimulates mTORC1/S6K1 activity, but rather than sustaining progenitor renewal, the result is glomerular hypertrophy and fibrotic signaling. Simultaneously, testosterone enhances calcineurin/NFAT activation, amplifying pro-inflammatory transcription and accelerating glomerulosclerosis [9, 10]. This dual effect helps explain the well-documented male disadvantage in CKD progression.

Kidney transplantation introduces a pharmacologic environment that inadvertently neutralizes both estrogen-mediated protective axes. Calcineurin inhibitors (CNIs), the backbone of transplant immunosuppression, uniformly suppress NFAT signaling. In men, this mimics estrogen’s inhibitory effect; in women, it represents a redundant blockade that abolishes a uniquely protective pathway. Simultaneously, mTOR inhibitors directly counteract estrogen-driven progenitor proliferation, eliminating the regenerative advantage observed in female kidneys. Thus, transplantation creates an artificial biological equivalence not by enhancing male resilience, but by pharmacologically suppressing female resilience.

This framework suggests that sexual dimorphism in transplant outcomes is not inherently absent but rather masked by immunosuppressive strategies that converge on hormone-sensitive pathways. Importantly, registry studies increasingly suggest sex-specific differences in transplant benefit and long-term outcomes, supporting the biological plausibility of this interaction.

We propose that future transplant studies should systematically stratify outcomes by sex, hormonal status, and immunosuppressive regimen. Tailoring mTOR inhibition or adjusting its timing may preserve regenerative capacity in women without compromising graft protection. By recognizing that women may lose their intrinsic regenerative advantage under current regimens, the field can move toward truly individualized and sex-specific immunosuppression strategies that protect graft survival without disrupting inherent biological strengths.

## Data Availability

The original contributions presented in the study are included in the article/supplementary material, further inquiries can be directed to the corresponding author.
